# Regioselective biooxidation of (+)-valencene by recombinant *E. coli *expressing CYP109B1 from *Bacillus subtilis *in a two-liquid-phase system

**DOI:** 10.1186/1475-2859-8-36

**Published:** 2009-07-10

**Authors:** Marco Girhard, Kazuhiro Machida, Masashi Itoh, Rolf D Schmid, Akira Arisawa, Vlada B Urlacher

**Affiliations:** 1 Institute of Technical Biochemistry, Universitaet Stuttgart, Allmandring 31, 70569 Stuttgart, Germany; 2 Bioresource Laboratories, Mercian Corporation, 1808 Nakaizumi, Iwata, Shizuoka 438-0078, Japan

## Abstract

**Background:**

(+)-Nootkatone (**4**) is a high added-value compound found in grapefruit juice. Allylic oxidation of the sesquiterpene (+)-valencene (**1**) provides an attractive route to this sought-after flavoring. So far, chemical methods to produce (+)-nootkatone (**4**) from (+)-valencene (**1**) involve unsafe toxic compounds, whereas several biotechnological approaches applied yield large amounts of undesirable byproducts. In the present work 125 cytochrome P450 enzymes from bacteria were tested for regioselective oxidation of (+)-valencene (**1**) at allylic C2-position to produce (+)-nootkatone (**4**) via *cis*- (**2**) or *trans*-nootkatol (**3**). The P450 activity was supported by the co-expression of putidaredoxin reductase (PdR) and putidaredoxin (Pdx) from *Pseudomonas putida *in *Escherichia coli*.

**Results:**

Addressing the whole-cell system, the cytochrome CYP109B1 from *Bacillus subtilis *was found to catalyze the oxidation of (+)-valencene (**1**) yielding nootkatol (**2 **and **3**) and (+)-nootkatone (**4**). However, when the *in vivo *biooxidation of (+)-valencene (**1**) with CYP109B1 was carried out in an aqueous milieu, a number of undesired multi-oxygenated products has also been observed accounting for approximately 35% of the total product. The formation of these byproducts was significantly reduced when aqueous-organic two-liquid-phase systems with four water immiscible organic solvents – isooctane, *n*-octane, dodecane or hexadecane – were set up, resulting in accumulation of nootkatol (**2 **and **3**) and (+)-nootkatone (**4**) of up to 97% of the total product. The best productivity of 120 mg l^-1 ^of desired products was achieved within 8 h in the system comprising 10% dodecane.

**Conclusion:**

This study demonstrates that the identification of new P450s capable of producing valuable compounds can basically be achieved by screening of recombinant P450 libraries. The biphasic reaction system described in this work presents an attractive way for the production of (+)-nootkatone (**4**), as it is safe and can easily be controlled and scaled up.

## Background

**Figure 1:**
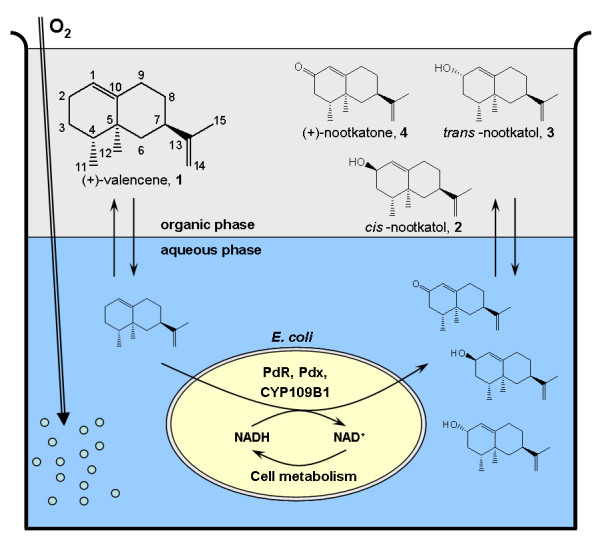
**Schematic experimental setup for the conversion of (+)-valencene (1) by CYP109B1 in a biphasic system**. (+)-Valencene **(1) **is converted to *cis*-nootkatol **(2)**, *trans*-nootkatol **(3) **and (+)-nootkatone **(4) **by recombinant *E. coli *expressing CYP109B1, putidaredoxin reductase (PdR) and putidaredoxin (Pdx). The organic phase acts as substrate reservoir and allows the accumulation of the mono-oxygenated conversion products, which prevents them from overoxidation.

The sesquiterpenes are a large class of terpenoid compounds and common constituents of plant essential oils. The parent sesquiterpenes are often readily available and their oxygenation gives derivatives which are sought-after fragrances and flavorings, pharmaceuticals or building blocks for chemical synthesis [1]. For example, (+)-valencene (**1**) is found in citrus oils and can be cheaply obtained from oranges. Recently the valencene synthase gene from orange has been cloned and functionally expressed in *E. coli *[2,3]. The selective oxidation of (+)-valencene (**1**) at allylic C2-position yields *cis*- (**2**) and *trans*-nootkatol (**3**), which can be further oxidized to (+)-nootkatone (**4**), a high added-value commercial flavoring (Figure 1). Traditionally nootkatone is extracted from grapefruits, and its price and availability is dependent on the annual harvest, which is restricted to a narrow producing area and very sensitive to weather conditions. Thus, various approaches for chemical or biotechnological production of (+)-nootkatone have been investigated in the past years (very recently reviewed by Fraatz et al. [4]).

Chemical synthesis of (+)-nootkatone (**4**) from (+)-valencene (**1**) has been studied with *tert*-butyl chromate [5], via copper(I)-mediated oxidation by alkyl hydroperoxides [6] and with surface-functionalized silica supported by metal catalysts such as Co^2+ ^and Mn^2+ ^[7]. However, these synthetic methods are neither safe nor environment-friendly, because they involve toxic heavy metals or peroxides.

Biotechnological processes to achieve this oxidation have also been designed. Recent attempts include the manufacturing of (+)-nootkatone (**4**) with green algae like *Chlorella *or *Euglena *[8], fungi such as *Aspergillus niger*, *Fusarium culmorum *[9], *Mucor *sp. [10,11] or the ascomycete *Chaetomium globosum *[12]. Cell free enzymatic reactions for the conversion of (+)-valencene (**1**) exploiting enzymes from *Cichorium intybus *L. roots [13], lignin peroxidase [14] and fungal laccase [15] have also been reported.

Several cytochrome P450 monooxygenases (P450s or CYPs) have been reported to catalyze terpene oxidation [16-21]. Cytochrome P450s belong to a superfamily of heme *b*-containing proteins that accept a vast number of organic compounds and catalyze an enormous variety of oxidative reactions. These reactions include hydroxylation, epoxidation, heteroatom oxidation, C-C bond cleavage via successive hydroxylations and many other complex reactions [22,23].

Interestingly, (+)-nootkatone (**4**) has been shown to inhibit activity of some human P450s, including CYP2A6 and CYP2C19 [24]. However, given the large superfamily of these enzymes (> 8100 sequences, ) and their diverse substrate range, it is unlikely that (+)-nootkatone (**4**) will inhibit all P450s. Indeed, a recent report describes the hydroxylation of (+)-valencene (**1**) by mutants of the cytochromes P450_cam _from *Pseudomonas putida *and P450_BM3 _from *Bacillus megaterium *[25]. In both cases, however, certain improvements are required. While the reported P450_cam_-mutants demonstrated quite low activity, the P450_BM3_-mutants, although being active, had low chemo- and regioselectivity and produced up to six byproducts besides nootkatol (**2 **and **3**) and (+)-nootkatone (**4**). Another report described a membrane-bound plant P450 monooxygenase from *Hyoscyamus muticus*, which was able to oxidize (+)-valencene (**1**) to nootkatol [16]. Generally, all current biotechnological systems for production of (+)-nootkatone (**4**) have in common an unsatisfactory accumulation of the C2-oxidized compounds because of low enzyme activity and/or the drawback of the formation of large amounts of byproducts.

In the search for new P450 monooxygenases with activity and high regioselectivity towards (+)-valencene (**1**), we screened 125 P450 enzymes from a novel recombinant P450 library, based on about 250 bacterial cytochrome P450 monooxygenases, co-expressed with putidaredoxin reductase (PdR) and putidaredoxin (Pdx) in *Escherichia coli*. The library contains, for example, the complete P450 complement of *Streptomyces coelicolor *A3(2), *Bacillus subtilis *168, *Nocardia farcinica *IFM 10152 and *Bradyrhizobium japonicum *USDA110. Generally, about 70% of the P450 candidates originate from actinomycetes. Construction and application of this P450 library has recently been reported with respect to steroid oxidation [26].

The actual screening identified CYP109B1 from *Bacillus subtilis*, which catalyzed the regioselective oxidation of (+)-valencene (**1**) to nootkatol (**2 **and **3**) and (+)-nootkatone (**4**). When the whole-cell biooxidation of (+)-valencene (**1**) with *E. coli *expressing CYP109B1 and electron transfer partners was carried out in an aqueous milieu, a number of undesired multi-oxygenated oxidation products was observed. In order to reduce byproduct-formation biphasic systems with water immiscible organic solvents were applied and the best system was scaled-up.

## Results and discussion

### Screening of the recombinant P450 library

**Figure 2:**
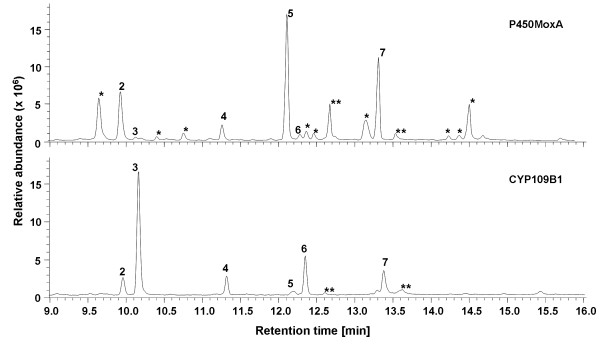
**GC diagrams of extracts of (+)-valencene (1) conversions**. Conversion of 2 mM (+)-valencene (**1**) was carried out with recombinant *E. coli *expressing PdR, Pdx and either P450MoxA or CYP109B1 in 2 ml aqueous CV2 buffer with 2% DMSO for 8 h at 30°C and extracted with ethyl acetate. Numbered peaks represent *cis*-nootkatol (**2**), *trans*-nootkatol (**3**), (+)-nootkatone (**4**) and overoxidation products (**5**, **6 **and **7**). * Conversion products of (+)-valencene (**1**) that could not be identified. ** Non-specific peaks that were also present in the negative control.

In our approach to find new P450s capable to oxidize (+)-valencene (**1**) regioselectively at allylic C2-position, 125 bacterial P450s from an *E. coli*-based recombinant P450 library were screened. Generally, P450 monooxygenases require electron transfer partners for activity. Since natural electron transfer partners for most of these 125 P450s are unknown, PdR and Pdx from *P. putida *were co-expressed in the *E. coli *cells. In the screening two enzymes were found to be able to oxidize (+)-valencene (**1**), namely CYP109B1 from *B. subtilis *168 and CYP105 from *Nonomuraea recticatena *NBRC 14525 (also referred to as P450MoxA [27]). The conversion of (+)-valencene (**1**) by CYP109B1 from *B. subtilis *resulted in six oxidation products, with the desired mono-oxygenated compounds nootkatol (**2 **and **3**) and (+)-nootkatone (**4**) accounting for approximately 65% of the total product, whereas oxidation of (+)-valencene (**1**) by P450MoxA yielded 15 different mono- and multi-oxygenated oxidation products (which mostly could not be identified), with the desired products accounting for less than 10% of the total product (Figure 2). The *E. coli *BL21(DE3) cells expressing only PdR and Pdx did not convert (+)-valencene. Because of its high regioselectivity, CYP109B1 was chosen as biocatalyst for further studies.

### Biooxidation of (+)-valencene by CYP109B1 in an aqueous system

Whole-cell biotransformation with *E. coli *BL21(DE3) cells expressing PdR-, Pdx- and CYP109B1 was first carried out in aqueous milieu (CV2 buffer, see Material and Methods). The average expression level of CYP109B1 verified through CO-difference spectra was 160 mg l^-1^, which corresponds to 7.6 mg g^-1 ^cell wet weight (cww). Since in all experiments 70 g cww l^-1 ^were used, the final P450 concentration was approximately 530 μg ml^-1^. Expression of PdR and Pdx was verified by sodium dodecyl sulphate polyacrylamid gel electrophoresis (SDS-PAGE) [see Additional file 1].

When (+)-valencene (**1**) was added directly to the cell suspension, the oxidation of the substrate by CYP109B1 proceeded with 270 nM min^-1 ^and after 8 h 6% conversion was achieved. Because of the low conversion, 2% dimethyl sulfoxid (DMSO) was added to the reaction. DMSO increases the solubility of (+)-valencene (**1**), which is poorly soluble in the aqueous reaction medium. In consequence the volumetric productivity could be increased up to 990 nM min^-1 ^and after 8 h 25% of the substrate was converted.

Electron transfer to CYP109B1 in *E. coli *cells can occur either via co-expressed heterologuos PdR and Pdx and/or via endogenous flavo- or ferredoxins and reductases from the host strain. To investigate, if co-expressed PdR and Pdx in fact support the activity of CYP109B1, *E. coli *BL21(DE3) was transformed with the pET28a-CYP109B1-plasmid comprising exclusively the CYP109B1-gene without PdR and Pdx (see Material and Methods section). In this case the biooxidation of (+)-valencene (**1**) preceded with only 40 nM min^-1^, regardless of the addition of DMSO, which demonstrates that co-expression of PdR and Pdx is necessary for activity of CYP109B1 *in vivo*.

For further evaluation of the electron transport to CYP109B1 via PdR and Pdx, a reconstituted *in vitro *system with recombinantly expressed and purified CYP109B1, PdR and Pdx [see Additional file 1] was set up as described in the material and methods section. In this system, the NADH oxidation rate was 64 nmol nmol^-1^ P450 min^-1^ and approximately 10% conversion of 200 μM (+)-valencene (**1**) was observed, however, only if all three proteins were added to the reaction mixture. Mixtures without PdR and/or Pdx did not show any activity towards (+)-valencene (**1**) (data not shown). Detailed biochemical characterization of CYP109B1 is a topic of our current investigations.

Gas chromatography (GC) analysis of reaction mixtures after the *in vivo *(+)-valencene (**1**) biotransformation revealed several new peaks besides the substrate peak (Figure 2). According to their mass fragmentation spectra (MS) and by comparison with authentic reference compounds three peaks were identified as *cis*-nootkatol (**2**) (retention time [RT] = 9.9 min), *trans*-nootkatol (**3**) (RT = 10.1 min) and nootkatone (**4**) (RT = 11.3 min) accounting for 6.2, 55.1 and 4.0% of the total product, respectively. Three other products were observed at RTs of 12.1, 12.3 and 13.3 min accounting for 2.3, 27.2 and 5.2% of the total product. A time course of the reaction demonstrated that these byproducts appeared shortly after the two nootkatol-isomers (**2 **and **3**) and nootkatone (**4**) were formed, which indicates that these compounds could represent products of overoxidation (data not shown). Furthermore, the fragmentation spectra and molecular weights of these compounds indicated the incorporation of two oxygen atoms into (+)-valencene (**1**) [see Additional file 2]. In a control experiment with nootkatone (**4**) as substrate instead of (+)-valencene (**1**), only the product with a RT of 13.3 min was identified by GC/MS analysis (data not shown). Obviously, this product is the oxidized nootkatone. Accordingly, when a mixture of nootkatol isomers (**2 **and **3**) was added as substrate, the products with RT of 12.1 and 12.3 min and small amounts of (+)-nootkatone (**4**) were observed. Thus these products were assumed to be products of further oxidation of the two nootkatol isomers (**2 **and **3**), which is in agreement with their MS data. In order to investigate if the primary oxidation products could be protected from overoxidation, we applied an aqueous-organic two-liquid-phase approach as shown in figure 1.

Remarkably, in the control experiment with nootkatone (**4**) instead of (+)-valencene (**1**), CYP109B1 oxidized approximately 60% of (+)-nootkatone (**4**) (final concentration 2 mM) within 2 h of conversion, whereas (+)-valencene (**1**) (final concentration 2 mM) was oxidized by only approximately 5% within this time. This means that nootkatone is actually a better substrate for CYP109B1 than valencene. Interestingly, this phenomena has also been reported for the P450_BM3 _wild type enzyme and mutants, which accept nootkatone as substrate much better than valencene [25].

### Biocompatibility of organic solvents

Aqueous-organic two-liquid-phase systems (further referred to as biphasic systems) represent a powerful biotechnological tool for biotransformations of toxic organic compounds [28-30]. This approach allows 1) the use of high overall concentrations of hydrophobic toxic substrates by regulating substrate and product concentrations in the aqueous biocatalyst phase, and 2) simple *in situ *product recovery into organic phase. Four water-immiscible organic solvents (*n*-octane, isooctane, dodecane and hexadecane) were chosen to set up biphasic systems and compared to the aqueous system. Control experiments showed that none of the organic solvents was oxidized by the host strain.

**Figure 3:**
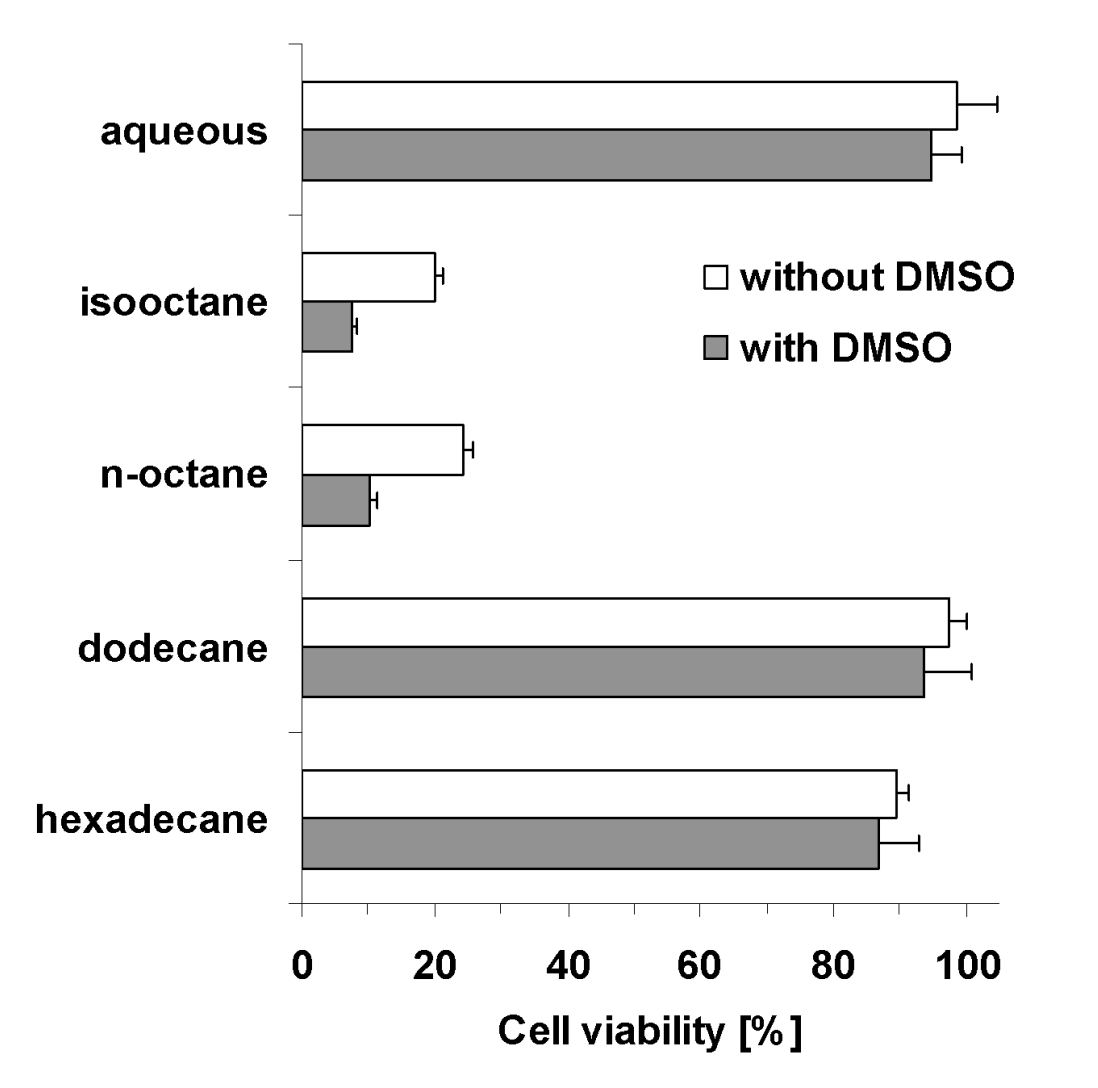
**Cell viability of *E. coli***. Cell viability of recombinant *E. coli *expressing PdR, Pdx and CYP109B1 was measured after 8 h exposure to biphasic systems consisting of CV2 buffer with or without 2% DMSO and 20% (v/v) organic solvents and compared to pure CV2 buffer (aqueous). Values represent mean ± standard deviation.

It is known that some organic solvents used as a second phase can damage the microbial cells [31]. Viability assays conducted after 8 h of (+)-valencene (**1**) conversion with *E. coli *expressing PdR, Pdx and CYP109B1 in systems with 20% (v/v) solvent showed that isooctane and octane had a strong effect on cell viability, with only 20% of cells surviving, whereas the more hydrophobic solvents dodecane and hexadecane were totally biocompatible with *E. coli*, as they did not harm the cell viability considerably. The addition of 2% DMSO had no considerable effect on cell viability in aqueous systems and in systems with dodecane or hexadecane, but led to further diminution of cell viability with octane and isooctane (Figure 3).

### Biooxidation of (+)-valencene (1) in biphasic systems

First, biphasic systems without addition of DMSO were set up. In these systems, however, the volumetric productivities were low (≤ 100 nM min^-1^) and after 8 h only 2% or less (+)-valencene (**1**) conversion was achieved in all systems tested. Presumably, the substrate holding capacity of the organic solvents was too high, preventing its oxidation in the aqueous phase. High distribution coefficients (log *D*) measured for (+)-valencene (**1**) in all four organic solvents confirm this observation (Table 1).

**Table 1:** Distribution coefficients for (+)-valencene (1), *trans*-nootkatol (3) and (+)-nootkatone (4)

Compound	DMSO [%]^a^	Distribution coefficient (log *D*)^b^			
		
		isooctane	*n*-octane	dodecane	hexadecane
(+)-valencene (**1**)	0	2.79 ± 0.03	2.61 ± 0.04	2.74 ± 0.05	2.62 ± 0.10
*trans*-nootkatol (**3**)	0	2.43 ± 0.03	2.31 ± 0.04	2.12 ± 0.02	2.11 ± 0.04
(+)-nootkatone (**4**)	0	2.63 ± 0.04	2.55 ± 0.03	2,68 ± 0.08	2.37 ± 0.12

(+)-valencene (**1**)	2	0.87 ± 0.05	0.18 ± 0,03	0.32 ± 0.08	0.62 ± 0.02
*trans*-nootkatol (**3**)	2	1.55 ± 0.05	1.57 ± 0.05	1.39 ± 0.06	1.23 ± 0.04
(+)-nootkatone (**4**)	2	1.77 ± 0.04	1.98 ± 0.05	1.83 ± 0.07	1.29 ± 0.06

^a^Final concentration in the mixture.

^b^Values represent mean ± standard deviation.

**Figure 4:**
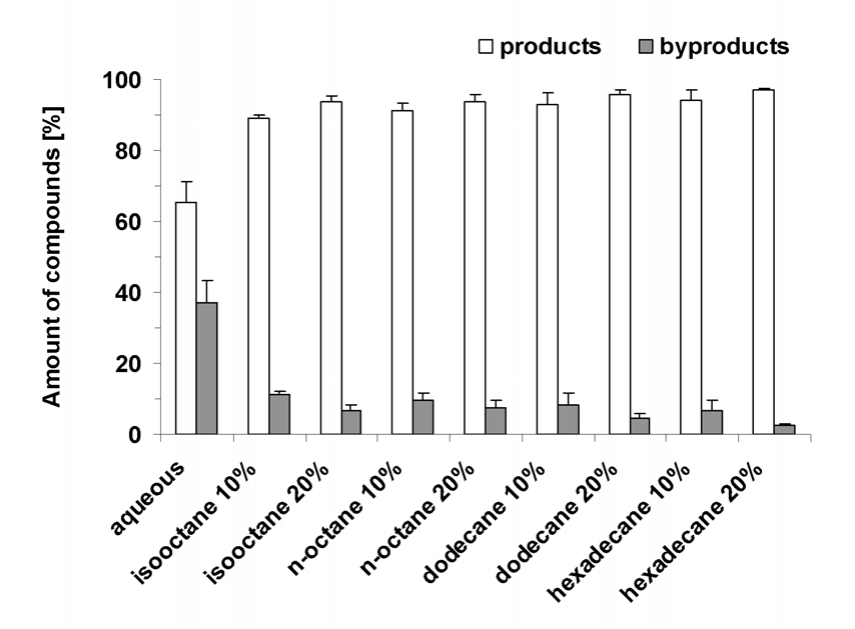
**Product distributions of (+)-valencene (1) conversions in biphasic systems**. Conversion of 2 mM (+)-valencene (**1**) was carried out with recombinant *E. coli *expressing PdR, Pdx and CYP109B1 in biphasic systems consisting of 2 ml CV2 buffer, 2% DMSO and organic solvents (v/v) as indicated for 8 h at 30°C and extracted with ethyl acetate. Values represent mean ± standard deviation, n ≥ 3. "Products" sum up the percental amounts of the total product of the two nootkatol isomers (**2 **and **3**) and (+)-nootkatone (**4**), "byproducts" combine the percental amounts of the total product of the compounds with RT 12.1, 12.3 and 13.3 min, respectively.

After addition of 2% DMSO the conversion of (+)-valencene (**1**) rose significantly in all biphasic systems employed, but was still lower in comparison to the aqueous system where 25% conversion was reached (Table 2). Generally, the addition of an organic phase led to a strong reduction of the generated multi-oxygenated byproducts causing a shift of the product distribution towards the mono-oxygenated compounds nootkatol (**2 **and **3**) and (+)-nootkatone (**4**) (Table 2). Moreover, the amount of byproducts decreased with increasing amounts of organic solvents from 10 to 20%. This tendency was similar for all four solvents tested. Basically, addition of 10% organic solvent reduced the amount of byproducts to less than 12% of the total product; 20% organic phase resulted in less than 7% byproducts. The best result in terms of reduction of byproducts was achieved with 20% hexadecane, were nootkatol (**2 **and **3**) and (+)-nootkatone (**4**) accounted for 97% of the total product (Figure 4).

**Table 2:** Product distribution and conversion of (+)-valencene (1) by CYP109B1 in biphasic systems^a^

Biphasic system	*cis*-nootkatol (**2**)^b^	*trans*-nootkatol (**3**)^b^	(+)-nootkatone (**4**)^b^	RT 12.1 min^b^	RT 12.3 min^b^	RT 13.3 min^b^	Conversion [%]^c^	Volumetric productivity [nM min^-1^]
aqueous	6.2	55.1	4.0	2.3	27.2	5.2	24.7 ± 3.8	990 ± 158
10% dodecane	7.4	82.8	2.6	---	6.1	1.1	19.5 ± 2.9	859 ± 121
20% dodecane	7.6	85.6	2.7	---	3.9	0.2	17.2 ± 1.8	719 ± 75
10% *n*-octane	7.7	80.2	3.2	0.4	7.3	1.2	16.4 ± 2.7	770 ± 116
20% *n*-octane	8.5	81.3	3.8	---	5.3	1.1	14.4 ± 2.4	502 ± 101
10% isooctane	10.1	74.9	4.1	0.1	8.5	2.3	9.6 ± 1.6	399 ± 68
20% isooctane	11.3	78.3	4.5	---	4.6	1.3	7.8 ± 1.8	313 ± 84
10% hexadecane	10.8	80.4	2.8	---	5.3	0.7	15.1 ± 3.5	631 ± 150
20% hexadecane	12.6	81.7	3.0	---	2.7	---	9.3 ± 1.2	387 ± 53

^a^Conversion of 2 mM (+)-valencene (**1**) was carried out with recombinant *E. coli *expressing PdR, Pdx and CYP109B1 in 2 ml CV2 buffer with 2% DMSO for 8 h at 30°C and extracted with ethyl acetate. All values are shown as mean (± standard deviation), n ≥ 3.

^b^Values are given in % of the total product.

^c^Percental amount of (+)-valencene (**1**) that was converted to products.

RT: Retention time during GC (for unidentified products).

---: Compound was not observed in the reaction. The detection limit was 4 μM.

Generally, the volumetric productivities of the biphasic systems were lower than those of the aqueous system (990 nM min^-1^). For example, addition of 10% of isooctane resulted in a 60% loss in original volumetric productivity (400 nM min^-1^), 20% of isooctane – in an almost 70% loss (Table 2). This high reduction is obviously due to the toxicity of isooctane to *E. coli*, as described in the previous section. For *n*-octane – which is toxic for *E. coli *as well – volumetric productivities of 770 and 502 nM min^-1 ^were obtained at 10% or 20% *n*-octane, respectively. Dodecane had the slightest effect on volumetric productivity; about 87% could be retained in the system with 10% dodecane after 8 h conversion (860 nM min^-1^). Interestingly, the retained volumetric productivities with hexadecane were significantly lower then those observed in the presence of *n*-octane, especially at 20% (Table 2), although the viability assay demonstrated that hexadecane is benign for *E. coli*.

When we determined the log *D *of (+)-valencene (**1**), *trans*-nootkatol (**3**) and (+)-nootkatone (**4**) for the biphasic systems with 20% organic solvent in the presence of 2% DMSO (Table 1), in the biphasic system with hexadecane a log *D *of 0.62 for (+)-valencene (**1**) was obtained, which is two-fold higher than with dodecane (0.32) and 3.5-times higher than with *n*-octane (0.18). The high log *D *for hexadecane implies a larger substrate holding capacity of this solvent, which might explain the significantly reduced volumetric productivity of the whole-cell process for this solvent compared to dodecane. The highest log *D *for (+)-valencene under these conditions was determined in isooctane (0.87), presumably also contributing to the low volumetric productivities in addition to the toxicity of this solvent.

Taking into account both, the reduction of byproduct formation and volumetric productivities, the best performance was observed in the biphasic system comprising 10% dodecane and 2% DMSO.

### Reaction scale-up

Conversion of (+)-valencene (**1**) was carried out in 20 ml volume for the biphasic system comprising 10% dodecane and 2% DMSO. The influence of cell density and substrate concentration on the yield of nootkatol (**2 **and **3**) and (+)-nootkatone (**4**) was investigated.

At a constant cww of 70 g l^-1 ^(corresponding to 18.4 g l^-1 ^cell dry weight (cdw)) higher substrate concentrations resulted, as expected, in higher overall amounts of desired products, however the substrate conversion values decreased (Table 3). For example, from 409 mg l^-1 ^(+)-valencene (**1**) 61.6 mg l^-1 ^nootkatol (**2 **and **3**) and (+)-nootkatone (**4**) were produced, and from 818 mg l^-1 ^(+)-valencene (**1**) – 96.0 mg l^-1 ^(Table 3). Product distribution did not change, independently on substrate concentration.

**Table 3:** Yields of nootkatol (2 and 3) and (+)-nootkatone (4) in biphasic systems with 10% dodecane^a^

Cww [g l^-1^]	c_valencene _[mg l^-1^]	Conversion [%]^b^	Yield [mg g^-1 ^cdw]	Yield [mg l^-1^]
70	409	15.1	3.35	61.6
70	613	13.2	4.40	80.9
70	818	11.8	5.22	96.0
140	409	21.8	2.42	89.1
280	409	23.0	1.28	94.2
140	818	14.1	3.14	118.5

^a^Conversion of (+)-valencene (**1**) was carried out with recombinant *E. coli *expressing PdR, Pdx and CYP109B1 in 20 ml CV2 buffer with 2% DMSO for 8 h at 30°C and extracted with ethyl acetate. Cell wet weight (cww) was as indicated; 70 g cww correspond to 18.4 g cell dry weight (cdw). Values are shown as mean of two experiments.

^b^Percental amount of (+)-valencene (**1**) that was converted to nootkatol (**2 **and **3**) and (+)-nootkatone (**4**)

At a constant substrate concentration of 409 mg l^-1 ^and two-fold increased cell density (140 g l^-1^) the yield of desired products could be increased from 61.6 mg to 89.1 mg l^-1^. A four-fold higher cell density resulted in 94.2 mg l^-1 ^(Table 3). Thus, higher cell densities at a constant substrate concentration lead to higher substrate conversion and higher overall amounts of nootkatol (**2 **and **3**) and (+)-nootkatone (**4**), but lower product yields per g of cells.

Under optimized reaction conditions 120 mg l^-1 ^of desired products were produced. In comparison, the concentration of desired products in the aqueous system under the same conditions reached 83 mg l^-1^.

## Conclusion

It was demonstrated that the identification of new P450s capable of producing valuable compounds can basically be achieved by the screening of recombinant P450 libraries. The P450-library used in this study is easy to handle and allows high throughput screening of various substrates.

For the biooxidation of (+)-valencene (**1**) with *E. coli *expressing recombinant CYP109B1 from *Bacillus subtilis*, Pdx and PdR the use of organic solvents improved the accumulation of nootkatol (**2 **and **3**) and (+)-nootkatone (**4**). Although the addition of organic solvents reduced the volumetric productivity of the whole-cell process, the production of nootkatol is promising. Considering the accumulation of nootkatol (**2 **and **3**) it should be noted that a bottleneck reaction seems to be the oxidation of nootkatol (**2 **and **3**) to (+)-nootkatone (**4**). Here both, process and enzyme engineering could help to overcome this limitation. For example, CYP109B1 could be optimized for higher affinity and activity towards (+)-valencene (**1**) and nootkatol (**2 **and **3**) with the goal to achieve an increased productivities of the whole-cell process and higher amounts of (+)-nootkatone (**4**). Alternatively, a specific dehydrogenase could be expressed in the same *E. coli *host to oxidize nootkatol (**2 **and **3**) to (+)-nootkatone (**4**) and thereby regenerate NADH, which in addition would reduce metabolic stress for the host strain.

## Methods

### Enzymes and materials

Restriction endonucleases, T4 DNA ligase, *Pfu *DNA polymerase and isopropyl β-D-thiogalactopyranoside (IPTG) were obtained from Fermentas (St. Leon-Rot, Germany). (+)-Valencene and (+)-nootkatone were from Fluka (Buchs, Switzerland). Glucose-6-phosphate dehydrogenase from *Leuconostoc mesenteroides *(EC 1.1.1.49) and other chemicals, solvents and buffer components were purchased from Sigma-Aldrich (Schnelldorf, Germany).

### Molecular biological techniques

General molecular biology manipulations and microbiological experiments were carried out by standard methods [32]. Construction of pT7-camAB – a plasmid based on pET11a (Novagen, Darmstadt, Germany) and harboring the *camA *(PdR) and *camB *(Pdx) genes from *Pseudomonas putida *ATCC 17453 – has been described previously [33]. pT7NS-camAB was prepared by insertion of an *Nde*I-*Spe*I linker into pT7-camAB and plasmids for the expression of bacterial P450s were created by amplification of annotated P450-encoding genes from genomic DNA of various bacteria as described in [33]. The plasmid for co-expression of PdR, Pdx and CYP109B1 – pCYP109B1-camAB – was constructed by amplification of the CYP109B1-encoding *yjiB*-gene [GenBank:CAB13078] from genomic DNA of the *Bacillus subtilis *strain 168 [34] and by following ligation of the PCR fragment into pT7NS-camAB utilizing the restriction sites for *Nde*I and *Spe*I of the *Nde*I-*Spe*I linker. pCYP109B1-camAB comprises the genes under control of the IPTG-inducible T7 phage-promoter in an artificial operon in the following order: *yjib *(CYP109B1), *camA *(PdR), *camB *(Pdx).

pET28a-CYP109B1 – a vector for the expression of CYP109B1 alone, without PdR and Pdx – was constructed as follows: The *yjiB*-gene was amplified from pCYP109B1-camAB with the primers 5'-AATA*gctagc*ATGAATGTGTTAAACCGCCG-3' and 5'-TCG*ctcgag*TTACATTTTCACACGGAAGC-3'. The PCR was performed with *Pfu *DNA polymerase under the following conditions: 95°C for 4 min, 25 cycles of (95°C for 1 min, 56°C for 30 s, 72°C for 3 min), 72°C for 5 min. The PCR-product was purified, digested with endonucleases *Nhe*I and *Xho*I and inserted into the previously linearized expression vector pET28a(+) (Novagen, Darmstadt, Germany). The resulting DNA-construct encoded for N-terminally His6-tagged CYP109B1 under control of the IPTG-inducible T7 phage-promoter.

pET28a-camA – a vector for the expression of PdR – was constructed by PCR-amplification of the *camA*-gene from pT7NS-camAB with the primers 5'-GGAATTCCATATGAACGCAAACGAC-3' and 5'-GATCGAATTCTCAGGCACTACTCAG-3'. The PCR was done as described for pET28a-CYP109B1, the PCR-product was purified and digested with endonucleases *Nde*I and *Eco*RI and inserted into previously linearized expression vector pET28a(+). The resulting plasmid encoded for N-terminally His6-tagged PdR under control of the IPTG-inducible T7 phage-promoter.

The basic steps for the design of pET28a-camB – which incorporates the *camB*-gene under control of the IPTG-inducible T7 phage-promoter for expression of N-terminal His-tagged Pdx – were identical to those for construction of pET28a-camA, except for primers. The primers 5'-GGGAATTCCATATGTCTAAAGTAGTGTAT-3' and 5'-CCGGAATTCTTACCATTGCCTATCGGG-3' were used for PCR in this case.

### Protein expression and purification

Recombinant CYP109B1 was expressed under the following conditions: *E. coli *BL21(DE3) cells were transformed with pET28a-CYP109B1 and transformants were selected on LB-agar plates with kanamycin (30 μg ml^-1^). 400 ml LB supplemented with 30 μg ml^-1 ^of kanamycin were than inoculated with 2 ml overnight culture – grown from a single colony – and grown at 37°C and 180 rpm, until the optical density at 600 nm (OD_600_) reached approximately 1.0. 100 μM IPTG, 80 μg ml^-1 ^5-aminolevulinic acid and 0.1 μM FeSO_4 _were added and the culture was grown for another 19 h at 30°C and 140 rpm. Cells were harvested by centrifugation at 10,000 × g for 20 min, the supernatant was discarded and the cell pellet was resuspended in 10 ml purification buffer (50 mM TrisHCl, pH 7.5, 500 mM NaCl, 100 μM phenylmethanesulfonyl fluoride). Cells were lysed by sonification on ice (3× 1,5 min, 1 min intermission), cell debris was removed by centrifugation (35,000 × g, 30 min, 4°C), the soluble protein fraction was recovered and filtered through a 0,2 μm filter.

Recombinant expression of PdR and Pdx was achieved by transformation of *E. coli *BL21(DE3) with either pET28a-camA or pET28a-camB. Transformants were selected on LB-agar plates with kanamycin (30 μg ml^-1^). 400 ml LB with kanamycin were inoculated with 2 ml from an overnight culture – grown from a single colony – and grown at 37°C and 180 rpm, until the OD_600 _reached approximately 0.7. 100 μM IPTG were added and the culture was grown for 17 h at 25°C and 160 rpm. The soluble protein fraction was recovered as described for CYP109B1.

Purification of CYP109B1, PdR and Pdx was done by immobilized metal affinity chromatography (IMAC) with a Talon^® ^resin (7 ml bed volume). Protein lysates were applied to the column, which was pre-equilibrated with 5 column volumes of purification buffer. Non-specifically bound proteins were washed from the column with 4 column volumes of purification buffer with 5 mM imidazol, before the bound protein was eluted with purification buffer containing 100 mM imidazol. 5% Glycerol was added to the eluate and it was dialyzed two times against 2 l of 50 mM TrisHCl, pH 7.5, containing 5% glycerol, 100 μM phenylmethanesulfonyl fluoride and frozen at -20°C until use.

### Determination of protein concentration

The P450 expression levels were estimated using the CO-difference spectral assay as described previously [35,36] using ε_450–490 _= 91 mM^-1 ^cm^-1^.

The concentration of PdR was determined as the average of the concentration calculated from each of the three wavelength 378, 454 and 480 nm using extinction coefficients 9.7, 10.0 and 8.5 mM^-1 ^cm^-1 ^[37].

The concentration of Pdx was determined as the average concentration calculated from the two wavelength 415 and 455 nm using extinction coefficients 11.1 and 10.4 mM^-1 ^cm^-1 ^[37].

### *In vitro *activity reconstitution of CYP109B1

A reconstituted *in vitro *system for conversion of (+)-valencene (**1**) by CYP109B1 was set up as described in [25] except for addition of catalase. The components were mixed in a 1,5 ml reaction tube, incubated at 30°C for 2 min and 200 μM NADH were added. Absorption at 340 nm was followed spectro-photometrical and NADH-consumption was calculated using ε = 6.22 mM^-1 ^cm^-1^. For product identification the setup was slightly modified as follows: 1 μM PdR, 10 μM Pdx, 1 μM CYP109B1, 5 units of glucose-6-phosphate dehydrogenase, 4 mM glucose-6-phosphat and 1 mM MgCl_2_. After incubation, the internal standard (-)-carvone (50 μM) was added, samples were extracted with 400 μl ethyl acetate and the extracts were analyzed by GC/MS.

### Construction of whole-cell biocatalysts

*E. coli *BL21(DE3) was used as a host for the gene expression. The BL21(DE3) cells were correspondingly transformed with plasmids harboring P450-genes and grown in 50 ml of M9 expression-medium, which is based on M9 medium [32], supplemented with 1% casamino acids, 20 μg ml^-1 ^thymine, 0.1 μM FeSO_4_, 80 μg ml^-1 ^5-aminolevulinic acid and 50 μg ml^-1 ^carbenicillin. For protein expression the Overnight Express™ Autoinduction System 1 (Novagen, Darmstadt, Germany) was added according to the supplier's manual. The cultivation was carried out for 24 h at 25°C. Cells were collected by centrifugation (2400 × g) and resuspended in 10 ml of aqueous CV2 buffer (50 mM potassium phosphate, pH 7.5, 2% glycerol, 0.1 mM IPTG, 50 μg ml^-1 ^carbenicillin), whereupon the cww was adjusted to 70 g l^-1^. The substrate was added to a final concentration of 2 mM for the biooxidation assay either directly or from a stock solution dissolved in DMSO to yield a final DMSO concentration in the reaction mixture of 2%. Samples were then split in 2 ml aliquots. For the biphasic systems 10 or 20% (v/v) of an organic solvent (isooctane, *n*-octane, dodecane or hexadecane) was added to the CV2 buffer. The biotransformation was carried out at 30°C under shaking at 200 rpm for 8 h. After incubation the internal standard (-)-carvone (50 μM) was added and samples were extracted with 1 ml of ethyl acetate for GC or GC/MS analysis. A negative control was performed with *E. coli *BL21(DE3) transformed with pT7NS-camAB.

For biooxidation reactions on a larger scale, 20 ml of CV2 buffer were set up with 70, 140 or 280 g_cww _l^-1 ^in round bottom flasks (70 g cww correspond to 18.4 g cdw). (+)-Valencene (**1**) was added from concentrated stock solutions in DMSO to achieve final concentrations of 2, 3 or 4 mM (+)-valencene (**1**) and 2% DMSO in the reaction mixture. The reaction was carried out at 30°C for 8 h under stirring with a magnetic stirrer. After incubation the internal standard (-)-carvone (50 μM) was added and samples were extracted with 10 ml ethyl acetate for GC analysis.

### Viability assays

*E. coli *viability during the biotransformation was monitored by taking cell aliquots from the reaction mixtures at certain time points. 40 μl of the aqueous phase were diluted in serial dilutions, plated on Luria broth agar plates containing ampicillin (100 μg ml^-1^) and after incubation at 30°C for 24 h the grown colonies were counted.

### Product analysis

The concentrations of (+)-valencene (**1**) and its conversion to products was determined by GC analysis using a GC2014 (Shimadzu, Kyoto, Japan) equipped with an Equity-5 column (30 m × 0.25 mm × 0.25 μm, Supelco, Pennsylvania, USA). The injector and detector temperatures were set at 250 and 270°C, respectively. 1 μl of a sample was injected at a split ratio of 4, with helium as carrier gas. The column temperature was maintained at 120°C for 4 min, ramped to 250°C at a rate of 10°C min^-1 ^and held at 250°C for 5 min. For quantitative GC analysis the FID response was calibrated for (+)-valencene (**1**) and (+)-nootkatone (**4**). Mixtures of CV2 buffer containing (+)-valencene (**1**) or (+)-nootkatone (**4**) in final concentrations of 50 to 2500 μM and (-)-carvone from a 5 mM stock solution in ethanol (final concentration 50 μM) as an internal standard were extracted with 1 ml of ethyl acetate and analyzed as described. The ratio of the area of the substrate to that of the internal standard was plotted against the substrate concentration to give a straight-line calibration plot.

Mass spectra were acquired on a GC/MS-QP2010 (Shimadzu) equipped with a FS-Supreme-5 column (30 m × 0.25 mm × 0.25 μm, Chromatographie Service GmbH, Langerwehe, Germany). The same setup as for the GC-analysis was used. The products were identified by their characteristic mass fragmentation patterns by comparison with mass spectra of authentic reference compounds and by comparison with mass spectra reported elsewhere [16].

### Determination of distribution coefficients

The distribution coefficients (log *D*) for (+)-valencene (**1**), *trans*-nootkatol (**3**) and (+)-nootkatone (**4**) were determined by dissolving increasing amounts of the respective compound (final concentration 500 to 5000 μM) in a 50% (v/v) mixture of aqueous CV2 buffer and an organic solvent (isooctane, *n*-octane, dodecane or hexadecane) in a total volume of 10 ml. After equilibration for 4 h at 25°C under vigorous shaking, phases were separated by centrifugation. 500 μl of each the organic and the aqueous phase were recovered separately and the internal standard (-)-carvone (final concentration 50 μM) was added. The aqueous phase was extracted with 1 ml of ethyl acetate and 500 μl of ethyl acetate were added to the organic phase. Both phases were analyzed quantitatively by GC. Subsequently the log *D *values were calculated.

### Synthesis of *cis*- (2) and *trans*-nootkatol (3)

390 mg (+)-nootkatone (**4**) was dissolved in 2 ml dry diethyl ether and added dropwise under stirring at 0°C on ice to a suspension of 130 mg LiAlH_4 _in 8 ml dry diethyl ether. After complete conversion, the solution was cooled down to -30°C and 20 ml saturated sodium potassium tartrate solution were added. The solution was allowed to warm to ambient temperature and was stirred for 16 h. The aqueous phase was extracted with diethyl ether (4 × 20 ml). The extracts were combined and dried over anhydrous MgSO_4_. Solvents were removed at 40°C. The raw product was applied to a silica gel column (ethyl acetate:petroleum ether 1:10). Fractions were analyzed by GC/MS and NMR. Nootkatol was isolated as a mixture of *cis*- (**2**) and *trans*-nootkatol (**3**) with a ratio of *cis*:*trans *of approximately 1:9. Solvents were removed from the nootkatol fraction at 40°C until no change in mass occurred. Nootkatol (**2 **and **3**) (350 mg, 89%) was retained as colorless oil.

^1^H-NMR (500 MHz, CDCl_3_, for numbering see figure 1): δ = 0.89 (3 H, d, *J *6.9, 4-Me), 0.95 (1 H, br s, 2-OH), 1.00 (3 H, s, 5-Me), 1.21 (1 H, dddd, *J *13.9, 12.4, 12.4, 4.3, 8-H_ax_), 1.37 (1 H, ddd, *J *12.7, 12.7, 10.0, 3-H_ax_), 1.52 (1 H, dqd, *J *13.0, 6.8, 2.1, 4-H), 1.71 (3 H, dd, *J *1.3, 1.1, 13-Me), 1.77 (1 H, dddd, *J *12.3, 2.3, 1.6, 1.3, 8-H_eq_), 1.78 – 1.84 (1 H, m, 3-H_eq_), 1.86 (1 H, ddd, *J *12.8, 2.7, 2.7, 6-H_eq_), 2.12 (1 H, ddd, *J *14.1, 4.2, 2.6, 9-H_eq_), 2.21 – 2.28 (1 H, m, 9-H_ax_), 2.29 – 2.37 (1 H, m, 7-H), 4.23 – 4.27 (1 H, m, 2-H_ax_), 4.67 – 4.70 (2 H, m, 2× 14-H_ax_), 5.32 (1 H, ddd, *J *2.6, 1.8, 1.8, 1-H_ax_) ppm.

^13^C-NMR (500 MHz, CDCl_3_): δ = 15.4 (4-Me), 18.2 (5-Me), 20.8 (13-Me), 32.4 (C-8), 32.9 (C-6), 37.3 (C-10), 38.2 (C-5), 39.3 (C-4), 40.8 (C-7), 44.6 (C-9), 68.0 (C-2), 108.6 (C-14), 124.3 (C-1), 146.1 (C-10), 150.2 (C-13) ppm.

## Competing interests

The authors declare that they have no competing interests.

## Authors' contributions

MG carried out the screening of the P450 library, the biphasic experiments and drafted the manuscript. VBU has made substantial contributions to conception and design of experiments and participated in writing the manuscript. AA, KM and MI participated in construction of the P450 library and interpretation of the screening results. All authors read the manuscript and gave final approval of the version to be published.

## Supplementary Material

Additional file 1 **12% SDS-PAGE of protein expression in recombinant E. coli and purified CYP109B1, putidaredoxin reductase (PdR) and putidaredoxin (Pdx)**. The picture provided shows protein expression as follows: Uninduced *E. coli *whole-cells (line 1); induced CYP109B1-, PdR- and Pdx-co-expressing strain (line 2); induced PdR- and Pdx-co-expressing strain (line 3); soluble protein fraction of CYP109B1 overexpression strain (line 4); purified CYP109B1 (line 5); purified PdR (line 6); purified Pdx (line 7); PageRuler™ Unstained Protein Ladder (line M). The molecular weights were estimated with 45.0 kDa for CYP109B1, 43.5 kDa for PdR and 11.6 kDa for Pdx. Click here for file

Additional file 2 **Mass spectra of oxidation products derived from (+)-valencene conversion**. The mass spectra provided correspond to the GC-chromatogram shown in Figure 2. Each spectrum is numbered according to the peak number given in Figure 2. The numbers represent spectra of *cis*-nootkatol (peak **2**), *trans*-nootkatol (peak **3**), (+)-nootkatone (peak **4**) and overoxidation products (peaks **5**, **6 **and **7**). Mass spectra of **2**, **3 **and **4 **were compared to those of authentic reference compounds that were either commercially available or have been synthesized chemically in our lab. Click here for file
