# Supplementary figures and images for: Regioselective biooxidation of (+)-valencene by recombinant E. coli expressing CYP109B1 from Bacillus subtilis in a two-liquid-phase system
Source: Microb Cell Fact. 2009 Jul 10;8:36. doi: 10.1186/1475-2859-8-36 (PMC2717049; doi:10.1186/1475-2859-8-36)

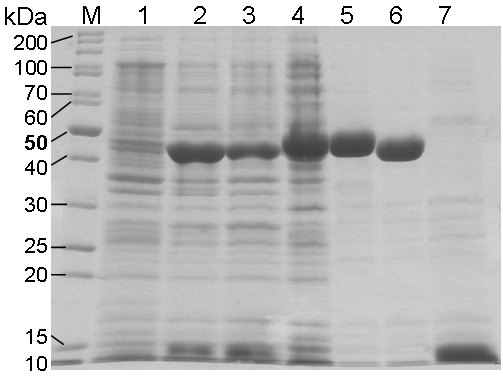

Supplement: Additional file 1 — 12% SDS-PAGE of protein expression in recombinant E. coli and purified CYP109B1, putidaredoxin reductase (PdR) and putidaredoxin (Pdx). The picture provided shows protein expression as follows: Uninduced E. coli whole-cells (line 1); induced CYP109B1-, PdR- and Pdx-co-expressing strain (line 2); induced PdR- and Pdx-co-expressing strain (line 3); soluble protein fraction of CYP109B1 overexpression strain (line 4); purified CYP109B1 (line 5); purified PdR (line 6); purified Pdx (line 7); PageRuler™ Unstained Protein Ladder (line M). The molecular weights were estimated with 45.0 kDa for CYP109B1, 43.5 kDa for PdR and 11.6 kDa for Pdx. [file 1475-2859-8-36-S1.png]

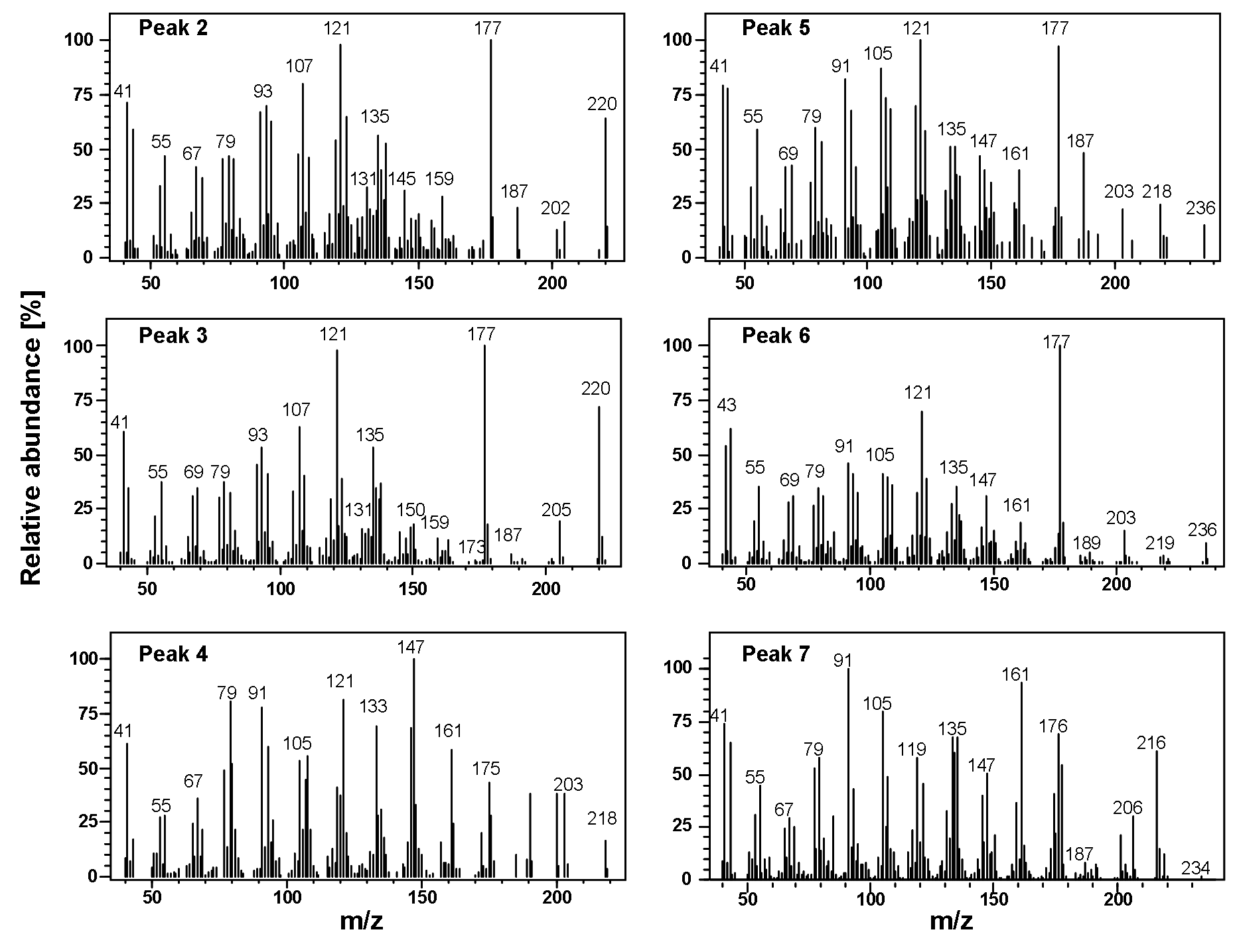

Supplement: Additional file 2 — Mass spectra of oxidation products derived from (+)-valencene conversion. The mass spectra provided correspond to the GC-chromatogram shown in Figure 2. Each spectrum is numbered according to the peak number given in Figure 2. The numbers represent spectra of cis-nootkatol (peak 2), trans-nootkatol (peak 3), (+)-nootkatone (peak 4) and overoxidation products (peaks 5, 6 and 7). Mass spectra of 2, 3 and 4 were compared to those of authentic reference compounds that were either commercially available or have been synthesized chemically in our lab. [file 1475-2859-8-36-S2.png]
